# MACSE: Multiple Alignment of Coding SEquences Accounting for Frameshifts and Stop Codons

**DOI:** 10.1371/journal.pone.0022594

**Published:** 2011-09-16

**Authors:** Vincent Ranwez, Sébastien Harispe, Frédéric Delsuc, Emmanuel J. P. Douzery

**Affiliations:** 1 Institut des Sciences de l'Evolution, UMR5554-CNRS, Université Montpellier II, Montpellier, France; 2 Centre de Recherche LGI2P de l'Ecole des Mines d'Alès, Nîmes, France; Texas A&M University, United States of America

## Abstract

Until now the most efficient solution to align nucleotide sequences containing open reading frames was to use indirect procedures that align amino acid translation before reporting the inferred gap positions at the codon level. There are two important pitfalls with this approach. Firstly, any premature stop codon impedes using such a strategy. Secondly, each sequence is translated with the same reading frame from beginning to end, so that the presence of a single additional nucleotide leads to both aberrant translation and alignment.

We present an algorithm that has the same space and time complexity as the classical Needleman-Wunsch algorithm while accommodating sequencing errors and other biological deviations from the coding frame. The resulting pairwise coding sequence alignment method was extended to a multiple sequence alignment (MSA) algorithm implemented in a program called MACSE (Multiple Alignment of Coding SEquences accounting for frameshifts and stop codons). MACSE is the first automatic solution to align protein-coding gene datasets containing non-functional sequences (pseudogenes) without disrupting the underlying codon structure. It has also proved useful in detecting undocumented frameshifts in public database sequences and in aligning next-generation sequencing reads/contigs against a reference coding sequence.

MACSE is distributed as an open-source java file executable with freely available source code and can be used via a web interface at: http://mbb.univ-montp2.fr/macse.

## Introduction

A wide range of molecular analyses rely on multiple sequence alignments (MSA), e.g., motif detection within genes and genomes [1], prediction of tridimensional structures [2], phylogenetic inference [3] and detection of positive selection [4]. In all these studies, the initial MSA can strongly impact conclusions and biological interpretations [5]. As a consequence, MSA is a richly developed area of bioinformatics and computational biology.

The DNA sequences to be aligned often contain open reading frames (ORF) that code for proteins. A coding sequence can be considered either at the nucleotide (NT) or amino acid (AA) level. Because of the redundancy of genetic codes, different codons encode the same AA. The NT sequence is thus less conserved but more informative than its AA translation. Since they are more informative, NT sequences should be able to provide equally good or even better alignments than their sole AA translation. In particular, aligning NT sequences may account for interrupted ORFs. These interruptions result from (i) the insertion of a non-multiple of 3 consecutive nucleotides – or the deletion thereof –, both inducing frameshifts that lead to transient or irreversible aberrant downstream AA sequence translation; and (ii) the substitution of an in-frame nucleotide resulting in unexpected, premature stop codons that shorten the AA sequence. These events may have either artefactual or biological causes. First of all, experimental errors may occur. Sequencing errors are frequent with the new sequencing technologies resulting in elevated error rates in homopolymers when using 454 GS-FLX [6] and in short read ends with Illumina Genome Analyzer [7]. This phenomenon is reinforced when ancient or present-day degraded DNA serves as PCR template [8]. Secondly, gene inactivation during the course of evolution leads to pseudogenes that exhibit disruption(s) of their original ORFs and whose identification has proven computationally difficult [9]. Thirdly, programmed frameshift mutations that are tolerated during translation have been widely documented [10] and their role in the evolution of novel gene function has been reported [11] To achieve higher NT alignment quality and detection of ORF interruptions, the AA translation should be taken into account during the alignment process. Ignoring it would mean omitting fundamental information. Yet, frameshifts and premature stop codons hamper the correct AA-guided alignment of NT sequences.

Numerous tools exist to align DNA sequences, among which are CLUSTAL [12], T-COFFEE [13], DIALIGN [14], MUSCLE [15], MAFFT [16], and the more recently proposed PRANK [3] and FSA [17]. However, when dealing with protein-coding sequences, these methods do not take into account the corresponding AA translations. Ignoring the AA translation is a major handicap in these methods for two main reasons [18], [19]: (i) as NT sequences are less conserved, clear similarities at the AA level can be obscured at the NT level thus complicating the alignment; (ii) current optimization criteria during the alignment procedure do not penalize insertion/deletion events (indels) that create translation frameshifts. As a result, a protein-coding sequence containing an insertion of two nucleotides followed by a downstream insertion of 7 nucleotides will have the same gap-related penalties as the more realistic scenario of an insertion of three nucleotides followed by another insertion of 6.

To overcome these problems, one common strategy consists of using a three-step approach. First of all coding NT sequences are translated into AA, these AA sequences are then aligned, and lastly, the obtained protein alignment is used for deriving the NT one. Tools such as revTrans [18], transAlign [19], PAL2NAL [20], and TranslatorX [21] were specifically developed to automate this straightforward alignment strategy. Note that PAL2NAL additionally allows to manually specify a priori the position of known frameshifts. DIALIGN [14] proposes this three-step strategy as an option for aligning DNA sequences. Moreover, it can either consider the full DNA sequence as coding, or search for its longest reading frame. The main drawback of this three-step approach is its inability to handle unexpected frameshifting substitutions. The AA translation that follows such events is no longer the correct one. At best, this erroneous translation will quickly lead to a stop codon that will alert the user and/or prevent the AA alignment. In other cases, the translated AA sequence will look like a highly divergent, orphan sequence at the protein level and will induce a partly aberrant DNA alignment. Such cases seem to be frequently encountered even in benchmark alignment datasets [22].

Unlike the vast literature on sequence alignment, few studies have focused on AA-aware NT sequence alignment. One of the first works on this subject was by Hein [23]. The author proposed a general DNA/protein model, where the cost of an alignment is a combination of its cost at the NT and AA levels. He then considered a special case where the two costs are simply summed and sequence evolution is idealized to involve only nucleotide substitutions and AA indels (no frameshift is allowed). An 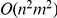 algorithm has been proposed to align two sequences of length 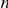 and 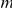 under this model [23]. A solution was then described to solve the same problem under affine gap costs in 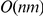 by Arvestad [24] and Pedersen et al. [25]. These improvements seemed to be promising as this algorithm reached the same asymptotic complexity as classical DNA alignment methods. However, the authors acknowledged that the constant factor masked by the 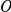 notation may be limitative in practice [25]. Indeed, to obtain a pairwise alignment, their method needs to compute 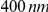 table entries which preclude its use in the MSA context.

An alternative approach that was recently proposed [26] consists of scoring the alignment according to a weighted sum of four costs: the NT alignment cost plus those of its three possible AA alignment translations. To make the algorithm simpler and faster, no specific cost is associated with indels that induce frameshifts. Here, frameshifting indels are supposed to be penalized by the AA mismatch they will induce. Considering all three reading frames may appear surprising since often only one is relevant, but this tool was specifically developed for handling viral genomes which may use overlapping reading frames [26].

In a slightly different context, an algorithm has been proposed to detect frameshift errors in newly determined NT sequences by comparison with AA sequences in public databases [27]. The algorithm generalizes the classical Smith-Waterman pairwise algorithm [28] so that the three reading frames are considered. An explicit frameshift cost is used to penalize frameshifts. This method provides an elegant solution for evaluating sequence proximity but cannot be extended to MSA since the underlying alignment cannot be displayed by the classical matrix representation used in MSA algorithms.

Here we present an AA-aware alignment algorithm where both input NT sequences could contain multiple frameshifts and/or stop codons. This pairwise coding sequence alignment method is fast enough to be extended to a MSA program called MACSE (Multiple Alignment of Coding SEquences). Indeed although pairwise solutions have existed for almost two decades, MACSE is the first MSA program able to align coding sequences based on their AA translations while accounting for frameshifts. We illustrate the relevance and usefulness of the MACSE program on biological case studies aimed at 1) computing MSA of protein-coding genes containing non-functional, pseudogene sequences, 2) aligning high-throughput sequencing reads against reference coding sequences and 3) detecting undocumented frameshifts in published sequences. MACSE is an efficient solution to detect errors in coding sequences and the first automatic solution to align pseudogenes while taking into account their potential AA translation and preserving their codon structure.

## Results

As illustrated in this section, MACSE is capable of producing an alignment of multiple protein-coding sequences possibly containing frameshifts and/or stop codons, either because these sequences contain errors or because they represent non-functional sequences. At the AA level, MACSE represents the stop codon by its usual symbol “*” and a codon containing a frameshift is represented by an extra symbol, the “!” (see figures below for examples). Meanwhile, at the nucleotide level, MACSE uses the symbol “!” to represent deletions of one or two nucleotides that induce frameshifts and it uses no special representation for the stop codon.

### Multiple alignment of functional and pseudogene sequences

Numerous evolutionary studies of individual genes or gene families involved in morphological adaptations require to quantify variation in selective pressure. Such analyses of molecular evolution based on codon models typically require aligning both functional and non-functional (pseudogene) sequences while respecting the underlying codon structure at the nucleotide level [4], [29], [30]. In this case, standard MSA programs that consider nucleotide sites independently disrupt the coding structure, while those that rely on AA translation are hampered by the presence of multiple frameshifts and premature stop codons.

**Figure 1:**
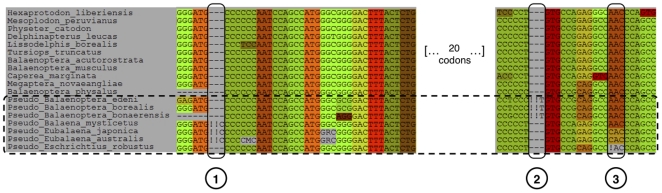
Open reading frame and pseudogene alignment of AMBN sequences in cetartiodactyls. Three situations are illustrated in which frameshifts detected by MACSE are indicated by exclamation marks. The 7 pseudogene sequences are boxed. Case 1: To maintain the reading frame, two exclamation marks are introduced in the *Balaena* and *Eubalaena* sequences. This pinpoints the occurrence of an extra C inserted in these three pseudogenes. Case 2: A similar situation in the three *Balaenoptera* sequences, with an extra T. Case 3: To maintain the reading frame, one exclamation mark is introduced in the *Eschrichtius* sequence. This pinpoints a single nucleotide deletion in this pseudogene. MACSE default parameters were used, i.e. matrix (BLOSUM 62), gap opening (−7), gap extension (−1), frameshift (−30), and stop codon (−100) except for pseudogene sequences for which lower penalties were assigned to frameshift (−20) and stop codon (−10).

As a first biological case, we show how MASCE can align multiple heterogeneous sequences from the *ambn* gene coding for ameloblastin. This enamel constitutive protein has been lost in whales whose teeth have been replaced by keratinous baleens [31]. In these species, the relaxation of selective constraints has allowed the accumulation of mutations leading to the occurrence of frameshifts and stop codons. Although no longer coding for a functional protein, the ghost of selection past acting on these pseudogenes nevertheless left traces of their former codon structure [32]. Using MACSE with the option adjusting frameshift and stop codon costs in pseudogenes rendered possible the incorporation of non-functional sequences in a codon-based alignment of functional orthologs of this gene (Fig. 1). Here, MACSE suggests the occurrence of three frameshifts, the positions of which are indicated by exclamation marks. In the first two cases they pinpoint the insertion of an additional nucleotide in several pseudogenes (Fig. 1: case 1 and 2) while in the third case a unique exclamation mark is introduced to indicate the probable deletion of a nucleotide in the pseudogene of *Eschrichtius* (Fig. 1: case 3).

**Figure 2:**
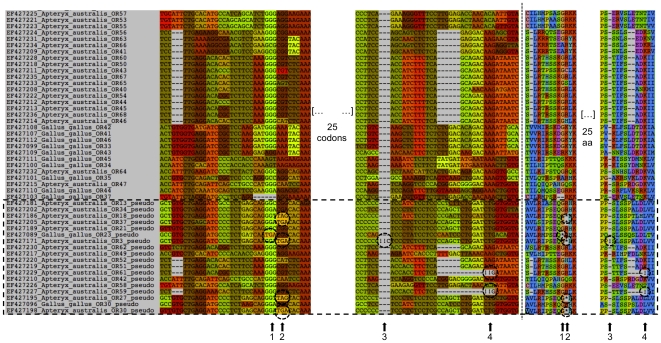
Snapshots of a multiple alignment of 93 functional and 18 pseudogene sequences from brown kiwi (*Apteryx australis*) and domestic chicken (*Gallus gallus*) olfactory repertoires. The same alignment region is displayed at the NT (left) and AA (right) levels. The 18 pseudogene sequences are boxed. Stop codons (stars in amino acid sequences) occurring at sites 1 and 2, and frameshifts (exclamation marks) inferred by MACSE at sites 3 and 4 are circled. MACSE guideline parameters for pseudogene datasets were used (see Fig. 1 for details.)

As a second example, we considered more divergent sequences from bird olfactory receptor genes. In this case, ecological differences among species have shaped the olfactory gene repertoires through gene duplication and pseudogenization events [29]. Here, we used MACSE to align 93 functional sequences with 18 pseudogenes from the brown kiwi (*Apteryx australis*) and domestic chicken (*Gallus gallus*) olfactory repertoires. The codon alignment highlights the occurrence of multiple stop codons (Fig. 2: sites 1 and 2) and the occurrence of frameshifts (Fig. 2: sites 3 and 4.) Stars and exclamation marks in the corresponding AA alignment respectively emphasize these events, which disrupt the coding frame while maintaining the correct translation. Note also that some functional sequences of these olfactory receptor genes share large in-frame deletions that are handled by MACSE.

Since MACSE alignments allow preserving the underlying codon structure they can be directly used to detect selection at the DNA level by estimating the dN/dS ratio with methods based on codon models of sequence evolution. Such analyses allow estimating where (along the gene) and when (along the phylogeny) pseudogenization events have occurred [4]. Note that other softwares (e.g., translatorX) were unable to align these datasets due to the presence of pseudogene sequences that display frameshifts. Hence, no matter which of the three possible reading frames is used the resulting translation contains stop codons. Indeed, pseudogene sequences should not be translated using a single reading frame as done by revTrans, transAlign or TranslatorX but using the three reading frames alternatively switching from one to the other at each frameshift. We also tested DIALIGN on these two case studies. The DIALIGN option searching for the longest reading frame is not satisfactory since sequences are truncated at the first encountered stop codon. Other DIALIGN options, including those based on AA translation, result in alignments that disrupt the codon structure by introducing numerous frameshifts and stop codons even in functional sequences. Finally, PAL2NAL might be used for this purpose but it requires specifying a priori the position of frameshifts in the AA alignment. By explicitly modeling frameshifting events and allowing distinct alignment penalties for different sets of sequences, MACSE has a main advantage over existing alignment tools, and is able to infer frameshift positions and propose more relevant alignments when non-functional sequences are sampled. This greatly facilitates subsequent analyses of molecular evolution based on codon models.

### Aligning raw sequences to a coding reference

With the exponentially growing DNA data generated by new high-throughput technologies, it has become particularly important to correctly align sequencing reads or contigs with the corresponding reference markers. Despite the high genome coverage generated by these approaches, the mapping and alignment tasks are complicated by the fact that 454 or Illumina reads may suffer from sequencing errors [6], [7]. Alignment-based methods have recently been proposed to correct sequencing errors in next-generation sequencing reads [33]. Since numerous phylogenomics and molecular evolution studies rely on expressed sequence tag (EST) data [34], MACSE can help computational biologists to align reads with their corresponding coding sequences.

As a second proof-of-concept example, we therefore illustrate the use of MACSE to align 454 reads obtained from a transcriptomic approach among mammalian rodents. There are five model rodents for which complete genome resources are available (*cf.* EnSEMBL v59): domestic mouse (*Mus musculus*), Norway rat (*Rattus norvegicus*), kangaroo rat (*Dipodomys ordii*), Guinea pig (*Cavia porcellus*), and ground squirrel (*Spermophilus tridecemlineatus*). Here, we focus on the transcriptome of a non-model rodent species, – the jerboa *Jaculus jaculus* –, belonging to the Dipodidae, a family which is closely related to Muridae including mouse and rat [35]. After gathering 454 reads from the jerboa transcriptome, we assigned them to the OrthoMaM collection of mammalian 1-to-1 orthologues [36] following a BLAST-based strategy.

**Figure 3:**
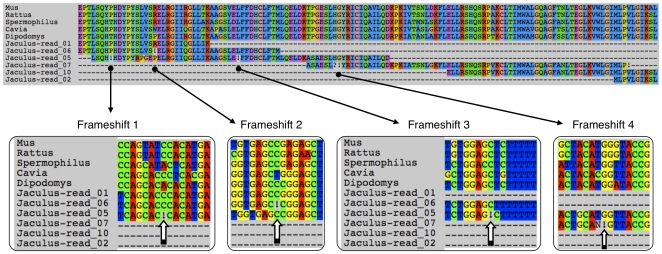
Alignment of 454 reads from a rodent transcriptome. Orthologues of the *tmem214* gene in 5 model rodents (mouse, rat, ground squirrel, Guinea pig, and kangaroo rat) were used as references to align 454 reads from the transcriptome of a non-model species, the jerboa (*Jaculus jaculus*). The MACSE protein alignment is given for the 5 model species and for 6 *Jaculus* reads. The insets focus on 4 regions in which frameshifts were detected. The corresponding nucleotide alignments are provided in a 15-site window. The exclamation marks suggest the location of sequencing errors in the coding sequence reads. MACSE default parameters were used, i.e. matrix (BLOSUM 62), gap opening (−7), gap extension (−1), frameshift (−30), and stop codon (−100) except for 454 reads for which lower penalties were assigned to frameshift (−10) and stop codon (−60).

In the case of the *tmem214* gene (EnsEMBL mouse accession ENSMUSG00000038828), several reads displayed problems. MACSE identified 4 frameshifts in 3 matching reads (Fig. 3). Detecting these frameshifts with MACSE will help contiging the reads, with procedures like CAP3 [37] or miraEST [38], especially in low-coverage regions for which less sequencing information is available to choose among alternative base calls. Moreover, if some reads concentrate frameshifts (see e.g. read_05 in Fig. 3), they can be a posteriori discarded from the subsequent assembly procedure. Finally, the error-free AA alignment produced by MACSE will be useful in phylogenomic studies relying on protein sequences for inferences of evolutionary relationships at deep taxonomic scales [39].

### Detecting frameshifts in coding sequences from public databases

**Figure 4:**
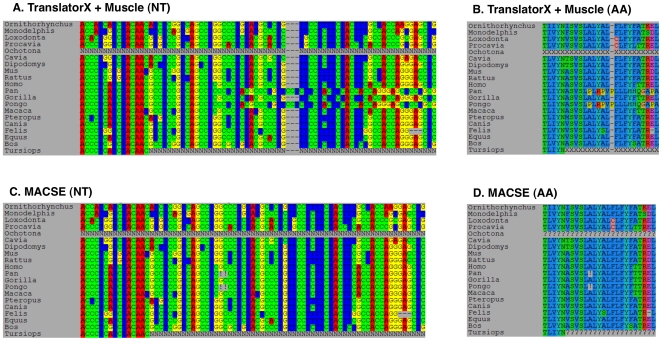
Alignments of orthologous CDS of the *tmem184a* gene (ENSG00000215155) from EnsEMBL v59. The TranslatorX+Muscle alignment is displayed at the nucleotide (NT) level (A) and at the Amino Acid (AA) level (B). Similarly, the MACSE alignment (obtained with default parameters) is displayed at the NT (C) and AA level (D).

As a last proof-of-concept example, we used the properties of MACSE to detect undocumented frameshifts in the EnsEMBL public sequence database [40]. During the construction of the OrthoMaM database of orthologous mammalian markers [36], we discovered a number of genes for which the sum of all branch lengths of the maximum likelihood phylogenetic tree is significantly departing from the average. To check whether this might be caused by undetected frameshifts in some of the coding sequences (causing them to be misaligned), we realigned these datasets using MACSE default options. Quite unexpectedly, several examples were revealed where some of the sequences indeed presented a shift in their reading frame induced by nucleotide indels. One striking example is provided by the *tmem184a* gene (ENSG00000215155) presented in Fig. 4.

Aligning the 1-to-1 orthologous coding nucleotide sequences of this gene with TranslatorX+MUSCLE (i.e. the AA alignment of TranslatorX is done with MUSCLE) resulted in an alignment where the chimpanzee (*Pan*) and orangutan (*Pongo*) sequences are clearly misaligned (Fig. 4A). This alignment error came from the AA translation of these sequences which resulted in highly divergent protein sequences (Fig. 4B). Applying MACSE to this dataset revealed that the two sequences in fact lack two nucleotides at the same site (Fig. 4C) resulting in a shift in their reading frame, in turn resulting in divergent AA sequences. As MACSE is able to efficiently detect these frameshifts, it returned correct alignments for both nucleotide and AA sequences (Fig. 4D) while indicating the most likely positions of those frameshifts in the sequences. Despite being guided by the AA translation of the sequences, TranslatorX is hampered by the fact that, by chance, these frameshifting indels do no lead to premature stop codons. By explicitly accounting for the underlying coding structure of the nucleotide sequences, MASCE is able to recognize that the most likely scenario is the presence of indels disrupting the coding frame. Whether the presence of these indels in curated coding sequences in a public database reflects annotation problems or sequencing errors is not known, but the problem may be more widespread than previously thought [22]. MACSE is a potentially efficient method for pinpointing and correcting such anomalies.

### Computing times

MACSE computation times remain reasonable compared to the human time spent aligning sequences that, up to now, no automatic method was able to align correctly. Though MACSE is slower than MUSCLE and TranslatorX+MUSCLE, MACSE is still a viable solution to align large datasets of hundreds of sequences and thousands of sites in a few computing hours. This section described several such examples where MACSE is worth the extra computation time. We also note that sequence alignment is often the first step in a long chain of analyses and that it may be worth investing time to obtain a reliable MSA before running, for instance, Bayesian phylogenetic inference which can require weeks of computation.

## Design and Implementation

### Model simplifications

Biological cases of disrupted reading frames are rare (e.g. in programmed frameshift mutations or pseudogenes) but sequencing errors that lead to apparent frameshifts are much more frequent. Such frameshifts occur through indels that are not multiples of three when one or two consecutive nucleotides are either deleted or inserted. To distinguish these kinds of frameshifts, we respectively denote as 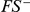 those induced by deletions, and by 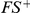 those induced by insertions. There are two main differences between our solution and other pairwise coding sequence algorithms (e.g. [23], [24], [26]). Firstly, our objective function is only based on sequence AA translations and secondly it ignores 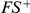 events. These two approximations allow us to extend our pairwise algorithm to MSA.

As mentioned in the introduction, Hein [23] and Pedersen et al [25] proposed defining the overall cost of the alignment as the sum of the costs of the two alignments. One can argue that the NT level is at least partly taken into account within classical AA substitution matrices such as PAM [41] or Blosum [42]. Using summation also raises the question of the relative importance of these two information levels in the alignment process since, as mentioned by the authors [25], other cost combinations could also be used. Hence, following the three-step strategy, we prefer to consider only the AA alignment cost which has the advantage of simplicity resulting in a faster solution.

Pairwise alignment algorithm accounting for frameshifts [24], [25], [27] explicitly model 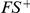 events (those representing the presence of one or two extra nucleotides in a sequence). Representing such events in the output alignment require either to remove the corresponding extra nucleotides from the sequence or to display it as a partial codon (e.g. “! ! C”) facing a “ghost” codon in the other sequence (“! ! !”) that is neither a real gap nor codon. None of these solutions is adapted to the classical strategy used to extend pairwise alignment algorithm to MSA (this strategy, based on alignment of alignments, is detailed at the end of this section). Removing the extra nucleotides prevents questioning this choice afterwards. Meanwhile, using a ghost codon (“! ! !”) is problematic, especially for correctly evaluating the costs of gap opening/closing when aligning two alignments. Indeed these costs are efficiently estimated based on the local configuration of gap and non-gap characters but since a ghost codon is neither one nor the other the standard solutions (e.g. [43], [44]) no longer work. This difficulty to handle 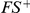 events is certainly the main reason for which previous pairwise solutions have never been extended to MSA. Note that ignoring 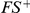 is not so dramatic since they can always be explained as a 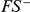 event in the concerned sequence facing a codon deletion in others (e.g. “! ! C” facing “– – –”). This is a practical approximation with little, if any, impact when only two sequences are aligned. In the case of MSA, this approach overpenalizes 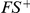 events (by adding deletions to other sequences), but it does not seem to have a major impact in practice. We acknowledge that an exact handling of 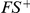 events would be preferable. Yet, as none have been found since Hein seminal work published in 1994, we think that it is time to consider approximate solutions to extend his pairwise model to a useful MSA tool.

### Defining the objective function of pairwise alignments containing frameshifts and stop codons

**Figure 5:**
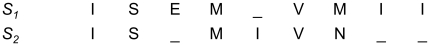
Simple pairwise AA alignment. This alignment describes a way to transform 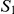 into 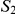 by deleting the E, inserting an I after the first M, changing the last M into an N, and deleting the two final I.

An alignment of two sequences 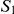, 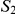 can be seen as a transformation process to turn 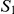 into 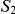 as illustrated in Fig. 5. Once a cost is associated with each elementary transformation (changing one letter into another, inserting/removing letters), the overall cost of the transformation process associated with an alignment can be computed by simply summing up the cost of its elementary transformations. An optimal alignment is then one with the minimum total transformation cost. To obtain a biologically meaningful alignment, the various elementary costs must be carefully chosen. The cost of turning one amino acid X into another Y depends on their physicochemical properties and is denoted as 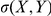. The cost of an insertion/deletion of 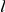 AAs is generally defined as 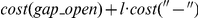 where 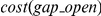 is a high value penalizing gap opening while 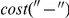 is a smaller value penalizing gap extension. This reflects the fact that indels are rare events (compared to substitutions) and that longer indels are even rarer. Note that this kind of gap cost is independent of the symbols that are inserted or deleted.

As explained above, our objective function only considers the AA alignment cost. From this point of view, it is sufficient to define the transformation cost related at the AA level to the two additional symbols used to represent frameshifting indels (“!”) and stop codons (“*”). Note that the probability of observing a frameshift or a stop codon in a sequence is relatively independent of what is observed in other sequences at the same site. The way to account for them is thus similar to the way indels are classically accounted for. Note that this is more than a coincidence for frameshift symbols since they indeed represent improbable indels of one or two nucleotides. The presence of “!” in front of any symbol is thus penalized with a high cost denoted as 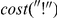. Similarly, the presence of “*” in front of any symbol has also a high cost denoted as 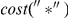. As a consequence, the presence of a “*” facing a “!” has a total cost of 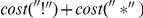.

Finally, stop codons appearing at the end of a sequence should not be penalized whereas frameshifting indels at sequence extremities must not be penalized more than other indels. From an algorithmic point of view, this is taken into account in our program in a way similar to indel costs that are generally handled to avoid penalizing those appearing at sequence ends.

### Finding the optimal alignment of two coding sequences with frameshifts and stop codons

Our solution, as most existing pairwise alignment methods of molecular sequences, is an improvement on the classical “Needleman-Wunsch” algorithm [45]–[47]. We thus start by recalling its basis. Having a sequence 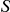, we denote 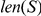 its length, and 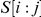 the subsequence of 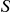 comprised between its 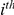 and 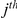 characters. Note that 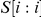 is thus the 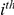 character of 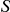 and that, by convention, 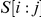 is the empty sequence (“”) if 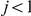 or 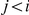. The first key observation is that the optimal alignment of two sequences can easily be deduced from the optimal alignments of the two sequences shortened by at most one character. More precisely, 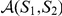 being the optimal alignment between two sequences 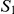 and 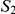 and its cost 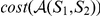, the overall cost of an optimal alignment between the two sequences can be recursively computed using the following formula (as long as 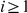 and 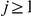):
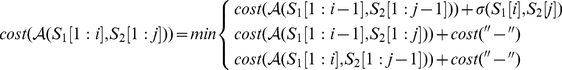
(1)


The recursion stops when at least one sequence is empty. An efficient solution for this recursive problem is to store each sub-problem solution. This only requires 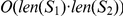 memory space while saving exponential computation time. The cost of each sub-problem solution is stored in a two-dimensional array of size 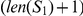
×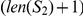 that we denote 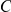 such that 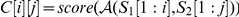. The first row and column of 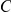 correspond to alignment containing an empty sequence with straightforward costs, e.g. 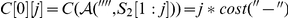. Once the first row and the first column are initiated, other cells of 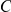 are considered in a left/right, top/down order. Hence each value of 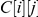 can be computed in constant time using the recursive formula (1) that relies on the three sub-problem costs stored in 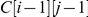, 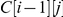 and 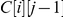. The last computed value (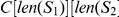) is the cost of an optimal alignment of 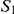 and 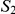. An optimal alignment can be obtained from the filled 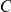 array by using a backtracking algorithm. This algorithm starts from the last entry of 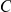 (i.e. 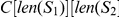) and determines which of its three neighbors has been used to obtain its optimal value. If the value comes from the left, it indicates an insertion of the last character of 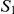; from the top, it is a deletion of this character; and from the diagonal, it is a substitution between the last two characters of 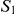, 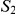. The algorithm then moves to the corresponding neighbor and the same process is repeated until the top left of the array is reached.

As we are looking for an alignment that takes into account the AA translation of the NT sequences, we need to introduce a new notation to link these two sequence levels. We will use 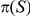 to denote the raw translation of a nucleotidic sequence 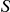 into AAs. This raw translation is realized using the first reading-frame, incomplete codons are converted into “!” and stop codons are converted into “*” without interrupting the translation. Considering two protein-coding nucleotide sequences without frameshifts 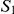 and 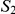, the 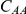 array used to align 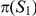 and 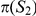 can be viewed as a compression of the corresponding 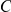 array that would have been used to align 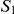 and 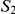. Indeed, each row (resp. column) of 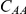 represents three rows (resp. columns) of 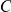. An alignment equivalent to the one produced by backtracking 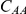 can thus be obtained using 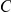 given that only movements corresponding to an AA substitution, insertion, or deletion are considered. These restrictions lead to considering only cells 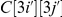 and to estimating their values based on the following formula (as long as 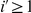 and 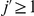):
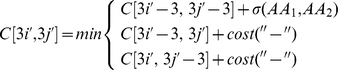
where 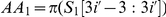 and 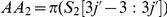.

**Figure 6:**
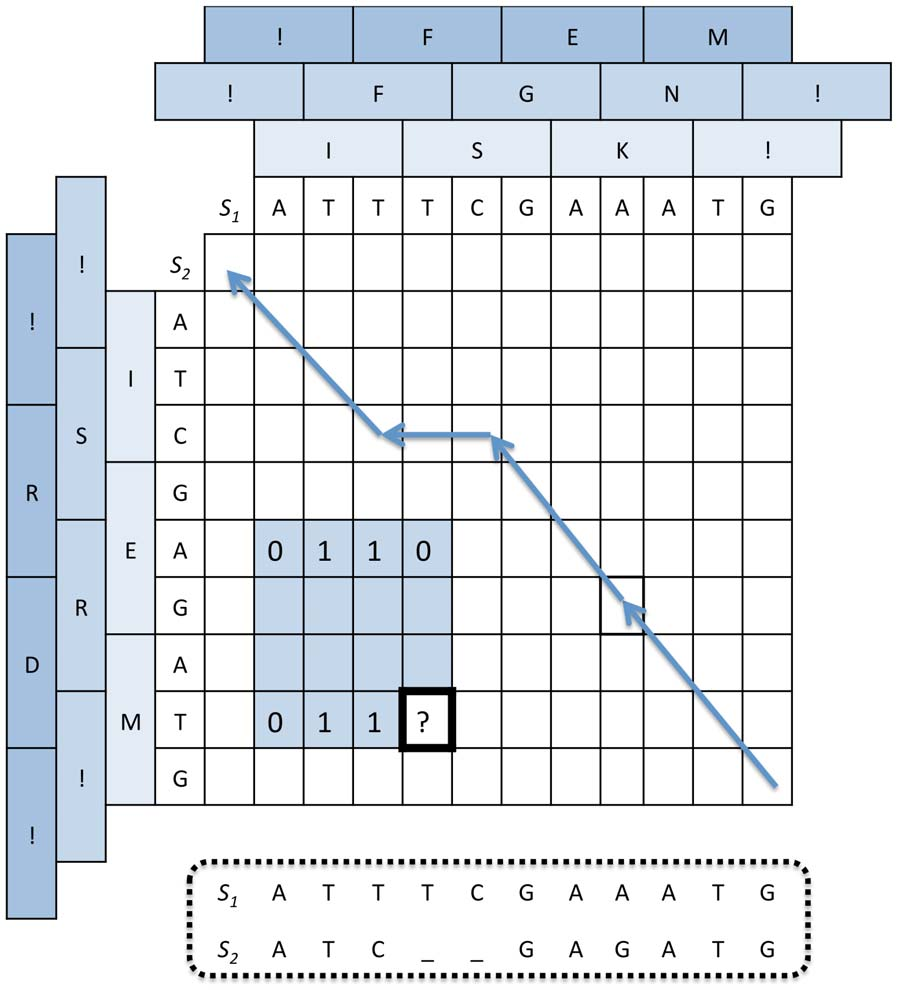
Alignment of two DNA coding sequences. Like for classical Needleman-Wunsch, an array is used to store the cost of an optimal alignment between prefixes of 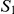
( = ATTTCGAAATG) and prefixes of 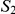
( = ATCGAGATG). The AA translations of those sequences are used to detect STOP codons and to evaluate codon substitutions based on their AA translations. The value of each cell is computed using 15 nearby cells. For instance, the bold cell value is computed based on its 15 colored neighbors. Among those 15 cells, some induced frameshifts in one or both sequences (see Fig. 7 for details). For instance cells marked with a “0” cause no frameshift, those marked by “1” cause a frameshift for 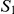 but not for 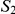. The optimal path (indicated by arrows) is determined using a backtracking process similar to the classical one, except that 15 possible moves are now considered. The alignment corresponding to this arrow path is depicted in the dashed box.

**Figure 7:**
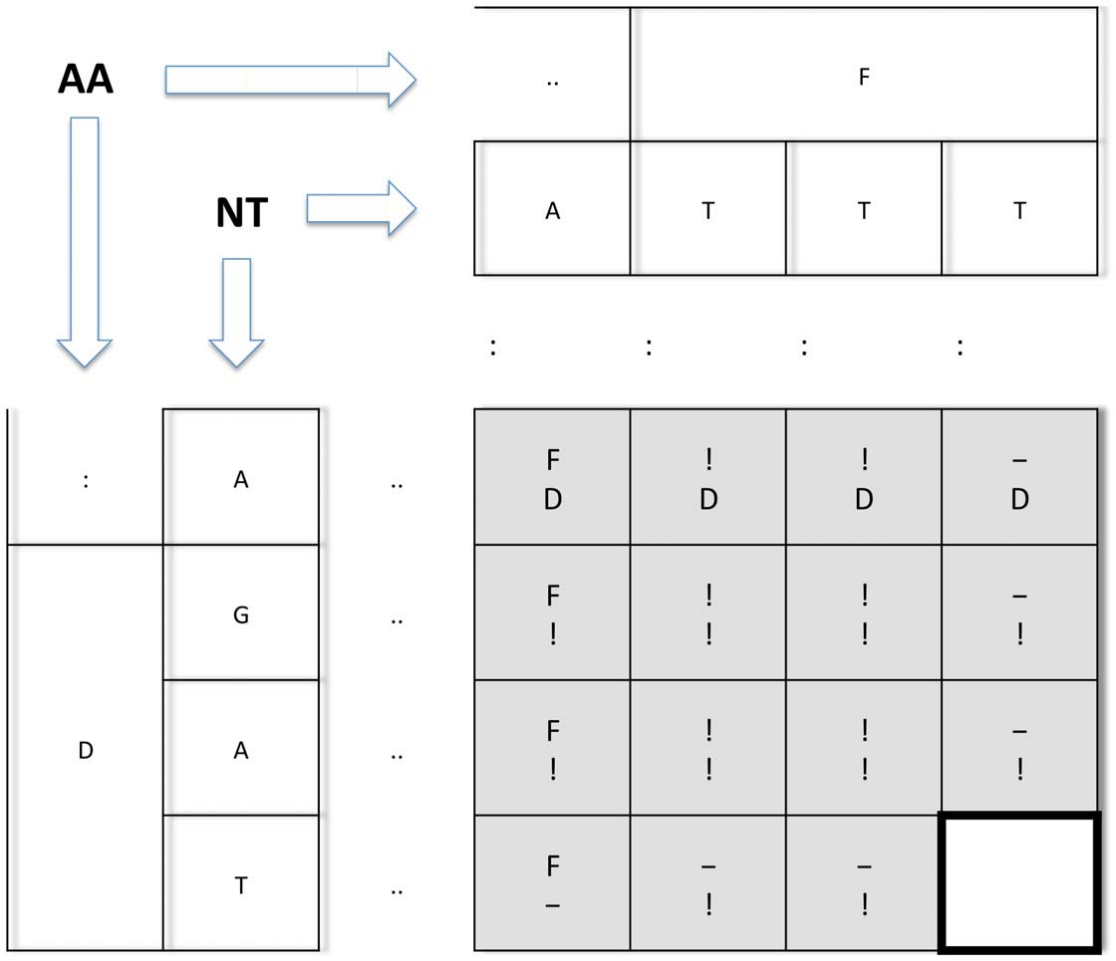
Relationship between the 15 possible moves and the proposed alignment. Suppose that the backtracking process has led to the bold cell. The next movement will go from this cell toward one of its 15 colored neighbors and one site will be added to the alignment constructed by the backtracking process. The site to be added is indicated for each cell.

Considering frameshift possibilities is a generalization of this approach where all cells 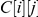 are considered and their values are estimated using all cells inside the square neighborhood delimited by 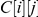, 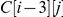, 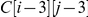 and 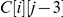. This 4×4 square thus defines 15 neighbor cells of 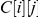 (Fig. 6). During the backtracking process, all movements from 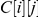 toward these 15 neighbors are considered. Three of them correspond to classical AA translations, while the 12 others induce 1 or 2 frameshifts. Fig. 7 shows the site alignments corresponding to these 15 possible movements. The resulting pairwise algorithm of two coding DNA sequences with respect to a frameshift and stop codon aware NT/AA model is detailed in Algorithm S1. Note that in this algorithm, values of 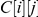 are accessed through a “get_C(i,j)” method that returns 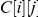 when 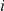 and 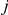 are valid indices, and 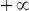 otherwise. The advantage is that the 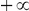 value does not interfere with the search for a minimum value, so that only the 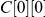 needs to be initialized while other cells in the three first rows (and columns) are handled like any others.

This dynamic programming algorithm is described using constant gap costs, i.e. the cost of an indel of size 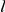 is just 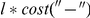. The implemented version is extended to handle the more realistic affine gap costs where the cost of an indel is 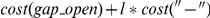. This is done by using three matrices 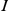, 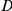 and 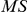 containing the optimal costs of partial alignment ending, respectively, by an Insertion, a Deletion or a Match/Substitution (e.g. [48]).

Since for each cell we consider 15 neighbors instead of the three considered in the standard Needleman-Wunsch algorithm, our approach is, theoretically, five times slower. Having a fast pairwise algorithm and a valid alignment representation, we can now apply classical MSA strategy based on this NT/AA model accounting for frameshifts and stop codons.

### Multiple alignment of protein-coding nucleotide sequences using an NT/AA model accounting for frameshifts and stop codons

A multiple alignment 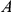 of 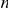 sequences 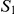,…,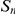 induces a pairwise alignment for any pair of sequences 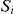, 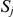 (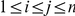) obtained by removing from 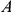 all other sequences and those sites that have a gap for both 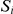 and 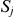. The cost of a multiple alignment is often defined as the sum of the cost of the pairwise alignment it induces. This criterion is called the sum-of-pairs (SP) score. Having two alignments 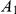 and 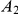 on disjoint sets of sequences 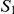 and 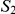, a variant of the dynamic programming algorithm used for two sequences allows an alignment 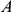 of 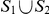 to be found, among those inducing 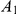 and 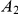, that has the lowest SP score. In this variant, a substitution cost is computed to reflect the sum-of-pairs criterion, i.e. it is a sum of elementary substitution costs for transforming AAs (resp. NTs) present in 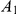 into those present in 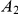. Gap extension costs can also be easily derived from the number of sequences included in both alignments, plus the gap frequencies of any of their sites. The only real difficulty is to correctly estimate the exact cost of gap creation that should be added to the SP score when considering an insertion/deletion event. Although this number can be computed exactly [44], the much easier way to compute “pessimistic gap count” estimation proposed by Altschul [43] appears to produce MSA of good quality [49].

The MSA produced by MACSE uses a progressive alignment strategy to obtain an initial draft MSA that is subsequently refined. Variants of this widespread strategy are used, for instance, by ClustalW [12], Muscle [15] and OPAL [49]. The influences of each step variant (such as the method used to measure sequence similarity) are extensively analyzed in the OPAL paper [49] and we considered its conclusions when designing MACSE. In particular, following their conclusions, we fixed the substitution matrix at BLOSUM62 [42]. The MSA strategy used in MACSE is obviously not the core of the present paper since we use the classical approach to extend our original pairwise alignment of coding sequences into a useful MSA. However, we briefly describe it below to explain the choice of our main variants.

Firstly, all pairwise sequence similarities are estimated based on the frequencies of their nucleotide k-mers, i.e. their sub-sequences of k nucleotides [50]. Those similarities are used to infer a dichotomic rooted guide tree using the UPGMA distance method [51]. By using UPGMA, the goal is clearly not to infer a phylogeny of the sequences but rather to build a guide tree that groups similar sequences, which must be aligned first [49]. The leaves of this tree are associated with the sequences to be aligned, whereas its internal nodes are associated with the MSA of the sequences included in the corresponding clade. The internal nodes are then processed bottom up, and the alignment of a node is obtained by aligning the previously computed alignments of its two descendants. Note that, following the conclusions of the OPAL paper, we choose to “align alignments” using the pessimistic gap count, as detailed in [48], rather than to align profiles, which is often the case e.g. [12], [15]. Since the profiles only consider the character frequencies of each site, they are less time and space consuming but do not contain enough information to compute gap cost according to the “pessimistic gap count”. The resulting MSA of the root node is then used as our initial draft of the desired MSA. We then use the classical 2-cut refinement strategy to improve it. This strategy consists of partitioning the current solution into two sub-alignments that are subsequently re-aligned. The resulting MSA replaces the previous one if its SP score is improved. This 2-cut refinement strategy also uses the guide tree: it iteratively considers each clade of the guide tree and splits the current global alignment so that one of the two sub-alignments contains the exact sequence of the clade concerned. Once all clades have been tested, a new guide tree is inferred using UPGMA based on sequence similarity estimated according to the sequence normalized contributions to the SP score of the current MSA [49]. Note that if the guide tree changes, some new 2-cut refinements will be tested. The refinement process stops when no more improvements are found, or when the maximum number of refinement iterations is reached.

### Availability, main features, and future directions

The MACSE program is distributed as an open source Java file executable with available source code. Since it is written in Java, MACSE is provided as a single jar file that works on every standard operating system (Windows, Linux, Mac OS). Once downloaded, it can be launched using the basic command line instruction e.g., “java -jar MACSE.jar -i my_seq.fasta -o my_output_prefix” (in the absence of any parameters, MACSE will print some help describing its options and providing some command line examples.) This allows to easily integrate MACSE in a bioinformatics pipeline. MACSE can also be used via a web interface at: http://mbb.univ-montp2.fr/macse.

### Main features and options of MACSE

MACSE takes input sequences in the FASTA format and provides as output two alignments of those sequences in the same format (one at the NT level and one at the AA level). The name of the input file and the basename to be used for the two output alignments are the only compulsory parameters of MACSE. One can easily define two sets of sequences that use different frameshift and stop codon costs by splitting sequences to be aligned into two different input files. This allows standard use cases to be handled when one wants to align either protein coding DNA sequences with pseudogenized ones, or curated sequences from public databases with sequences resulting from the raw output of new generation high-throughput sequencing technologies. The alignments outputted by MACSE can be examined using the SEAVIEW program [52], [53] which has a well suited codon view option.

The parameter values for gap opening/extension costs strongly influence the alignment produced by any MSA approach. Despite all efforts to design an automatic strategy to adjust these costs, the results obtained with such adjusted parameters are still disappointing compared to those that could have been obtained by the same MSA method if the true parameters were known [49]. The MACSE documentation includes some guidelines to choose cost penalties associated with gap opening/extension and with frameshift and internal stop codon occurrences for the most common usages – e.g. alignment of (pseudo)genes. Note also that since the user can provide an initial alignment that MACSE will use as a starting point for its 2-cut refinement strategy, one can rapidly test different parameter sets.

MACSE also integrates the alternative genetic codes, and provides options to specify the default genetic code to be used and/or to specify different codes to be used depending on sequence names. For the latter option, MACSE relies on a separate option file compatible with the one used by TranslatorX.

### Future directions

Future works include further optimization to speed up the program and the development of a more elaborated penalty model to take into account, for instance, the fact that frameshifts are more frequent within homopolymer portions of sequences. We also work on handling untranslated regions (UTR) that can appear at the beginning and/or end of the EST sequences. This can be done by adapting our algorithm to allow local alignment together with identification of start and stop codons at their extremities. Finally, we plan to collaborate with the SEAVIEW developer team to provide MACSE as a SEAVIEW plug-in.

## Supporting Information

Algorithm S1 Optimal pairwise computation cost for two coding DNA sequences.
(PDF)
