# Supplementary material for: MACSE: Multiple Alignment of Coding SEquences Accounting for Frameshifts and Stop Codons
Source: PLoS One. 2011 Sep 16;6(9):e22594. doi: 10.1371/journal.pone.0022594 (PMC3174933; doi:10.1371/journal.pone.0022594)
Supplement: Algorithm S1 — Optimal pairwise computation cost for two coding DNA sequences. (PDF) [file pone.0022594.s001.pdf]

Algorithm S1: Optimal pairwise computation cost for two coding DNA sequences

Data: Two Sequences S_1 and S_2, a *cost* method that returns the cost of frameshift, deletion and STOP codon, a σ method returning the AA substitution cost.

Result: An array C such that $C[i][j] = score(\mathcal{A}(S_1[1:i], S_2[1:j]))$.

for $i = 0$ **to** $len(S_1)$ **do**
  for $j = 0$ **to** $len(S_2)$ **do**
    if $i == 0$ AND $j = 0$ **then**
      C[i][j]=0;
    else
      $AA_1 =$ "?"; $AA_2 =$ "?";;
      if $i - 3 > 0$ **then** $AA_1 = (\pi(S_1[i-3:i])$;
      if $j - 3 > 0$ **then** $AA_2 = (\pi(S_2[j-3:j])$;

      stopS_1=0; stopS_2=0;
      if $AA_1 ==$ " $*$ " **then** stopS_1 = cost("*");
      if $AA_2 ==$ " $*$ " **then** stopS_2 = cost("*");

      if $AA_1 ==$ " $*$ " OR $AA_2 ==$ " $*$ " **then**
        subst_AA=stopS_1 +stopS_2
      else
        if $i - 3 > 0$ AND $j - 3 > 0$ **then**
          $subst\_AA = \sigma(AA_1, AA_2)$
        else
          $subst\_AA = +\infty$

$$C[i][j] = min \begin{cases} get\_C(i-3, j-3) + subst\_AA \\ get\_C(i-3, j) + stopS_1 + cost("\_") \\ get\_C(i, j-3) + cost("\_") + stopS_2 \\ \\ get\_C(i-3, j-2) + stopS_1 + cost("!") \\ get\_C(i-3, j-1) + stopS_1 + cost("!") \\ get\_C(i-2, j-3) + cost("!") + stopS_2 \\ get\_C(i-1, j-3) + cost("!") + stopS_2 \\ \\ get\_C(i, j-1) + cost("\_") + cost("!") \\ get\_C(i, j-2) + cost("\_") + cost("!") \\ get\_C(i-1, j) + cost("!") + cost("\_") \\ get\_C(i-2, j) + cost("!") + cost("\_") \\ \\ get\_C(i-1, j-1) + 2 * cost("!") \\ get\_C(i-1, j-2) + 2 * cost("!") \\ get\_C(i-2, j-1) + 2 * cost("!") \\ get\_C(i-2, j-2) + 2 * cost("!") \end{cases}$$

return C;
